# Defects of CTLA-4 Are Associated with Regulatory T Cells in Myasthenia Gravis Implicated by Intravenous Immunoglobulin Therapy

**DOI:** 10.1155/2020/3645157

**Published:** 2020-02-14

**Authors:** Wenhua Xu, Mingshan Ren, Swagata Ghosh, Kai Qian, Zhaofeng Luo, Aimei Zhang, Cuiping Zhang, Jiajun Cui

**Affiliations:** ^1^Department of Neurology, The First Affiliated Hospital of University of Science and Technology of China, Hefei, China; ^2^Department of Internal Medicine, Division of Infectious Diseases and International Health, University of Virginia, Virginia 22908, USA; ^3^The Center for Translational Medicine, Yichun University, Yichun, China; ^4^Hefei National Laboratory for Physical Science, Core Facility Center for Life Sciences, University of Science and Technology of China, Hefei, China; ^5^Central Laboratory, The First Affiliated Hospital of University of Science and Technology of China, Hefei, China

## Abstract

Myasthenia gravis (MG) is a CD4^+^ T cell-dependent autoimmune disease resulting from aberrant immune response mediated by circulating autoantibodies at the neuromuscular junction. Intravenous immunoglobulin (IVIg) is an expensive and commonly used immunotherapeutic approach to treat patients with MG. The mechanisms of actions involved in IVIg treatment, however, remain to be investigated. In an effort to examine the roles of various subsets of CD4^+^ T cells in the periphery blood of MG and uncover the mechanisms that contribute to the therapeutical effects of IVIg, we first demonstrated that a subset of CD4^+^ T cells, CTLA-4-expressing regulatory T (Treg) cells, were underrepresented and functionally defective in MG patients. The dynamic profiling during the IVIg therapy course further revealed an inverse relationship between the frequency of CTLA-4^+^ Treg and the quantitative MG (QMG) score that represents disease severity. Our mechanistic studies indicated that IVIg expands CTLA-4-Treg cells via modulating antigen-presenting dendritic cells (DCs). To determine the molecular defects of CTLA-4 in abnormities of Treg in MG patients, we demonstrated hypermethylation at -658 and -793 CpGs of *CTLA-4* promoter in MG Tregs. Interestingly, IVIg therapy significantly reduced the methylation level at these two sites in MG patients. Overall, our study may suggest a role of CTLA-4 in functionally defected Treg cells in MG and its actions involved in IVIg therapy.

## 1. Introduction

Myasthenia gravis (MG) is an autoimmune disorder characterized by varying degrees of muscle weakness and fatigue. It is mainly caused by autoantibodies against the postsynaptic acetylcholine receptors (AChRs) at the neuromuscular junction [1–3]. The synthesis of the pathogenic anti-AChR antibodies, which are primarily high-affinity IgG, requires the elicitation and intervention of CD4^+^ T cells, also called effector T cells (Teff), and their associated cytokines [4–6].

CD4^+^ T cells play central roles in the adaptive immune system. Naïve CD4^+^ T cells after being activated can be differentiated into a range of distinct lineages based on cytokine secretion patterns, including classical Th1 and Th2 cells, the more recently identified Th17 cells, follicular helper T (Tfh) cells, and regulatory T (Treg) cells [7–9]. Those CD4^+^ T cell subsets have been implicated in the development of a number of autoimmune diseases including MG [10–12]. IFN-*γ*-producing Th1 cells have been shown to be involved in the pathogenesis of many organ-specific autoimmune diseases [13]. Both Th1 and Th2 cells have been reported to be correlated with clinical signs of induced experimental autoimmune myasthenia gravis (EAMG) [14, 15]. The alterations of Th17 cells and their related cytokines, IL-17 and TGF-*β*1, have been observed in MG patients with thymomas [16]. Tfh cells provide help to B cells in germinal center and have been shown to exhibit an enhanced expression in the thymoma related to the clinical severity of MG [17, 18]. As the major regulator of T cell-mediated immunity, regulatory CD4^+^ T (Treg) cells express forkhead box P3 (FOXP3) transcription factor and suppress aberrant immune responses that result in autoimmune disorders [19–21]. Defects in FOXp3^+^ Treg cells have been shown to contribute to the development of MG and EAMG by us and many others [16, 22–26].

Although FOXp3 has been proposed as the master regulator that controls the suppressive function of Treg cells, a significant percentage of human-activated FOXp3-expressing T cells do not possess regulatory function, suggesting that other factors may operate concurrently with FOXp3 to mediate Treg function [27–30]. One of the candidate regulators is cytotoxic T lymphocyte-associated antigen-4 (CTLA-4). CTLA-4 is an inhibitory costimulatory factor constitutively expressed in a large portion of FOXp3^+^ Treg cells and has been shown to be critical to their suppressive function [31–33]. The aberrant expression of CTLA-4 has been observed in MG patients in a number of studies [34, 35]. However, whether the abnormalities of CTLA-4 contribute to Treg defects in MG and the role of CTLA-4 in the pathogenesis of MG are underappreciated.

Intravenous immunoglobulin (IVIg), a therapeutic preparation of pooled human polyclonal IgG, is a costly and frequently used immunomodulation therapy for patients with autoimmune and inflammatory diseases [36, 37]. Although IVIg therapy has been used for three decades, the mechanisms by which it benefits autoimmune patients are not completely understood.

In this study, we analyzed the peripheral blood derived from patients with MG before or after IVIg treatment in an attempt to identify defects in Treg that are involved in the pathogenesis of MG. We also analyzed other major lineages of CD4^+^ T cells including Th1, Th2, Th17, and Tfh given their pathologic significance in EMAG and MG [11]. The results indicated that Treg-associated CTLA-4 are underrepresented in MG patients and reduced expression and functional abnormalities in Treg-associated CTLA-4 are related to the disease severity of MG. We also showed that IVIg exerted its therapeutic effects by restoring Treg frequency and function through its effects on dendritic cells (DCs). Further, we uncovered that the abnormalities of CTLA-4 were associated with the methylation binding site within the CTLA-4 promoter, which could aid in the design of novel therapeutic strategies towards epigenetic regulation of CTLA-4.

## 2. Materials and Methods

### 2.1. Patients

This study enrolled 39 patients with myasthenia gravis (MG) and 59 age-matched healthy donors (HD) from March 2011 to May 2015. None of the patients had received any immunosuppressive therapy before enrollment. All patients signed an informed consent prior to their inclusion in the study. The study has been approved by the ethics committee of Anhui Medical University and conforms to the Declaration of Helsinki and its later amendments. The MG patients were diagnosed by neurologists according to the standard clinical criteria and divided into five subgroups according to the MGFA clinical classification [38]. The clinical characteristics of MG patients are summarized in Table 1 and the patient's numbers were 9 for class Ι, 16 for class II, 9 for class III, and 5 for class IV. There were no patients in class V. All MG patients' peripheral blood samples were subjected to autoantibodies test including AChR and MuSK antibodies. Three patients are with ocular muscle weakness. The other 36 patients were anti-AChR antibody positive, and one of them was also anti-MuSK antibody positive. The mean ± standard deviation (SD) of the ages was 39.34 ± 14.37 years in the MG patients and 39.00 ± 12.58 years in the healthy donors. The sex ratio (male : female) was 1 : 2 in the MG patients and 19 : 40 in the heathy donors. 20 of the 39 MG patients were suffering from progressive general weakness and received two cycles of intermittent intravenous immunoglobulin (IVIg) treatment with a dose frequency of 0.4 mg/kg/day for five consecutive days. 13 of them underwent thymectomy after IVIg therapy. A quantitative MG scoring system (QMG score) was applied to objectively assess the disease severity [38].

### 2.2. Isolation of Lymphocytes from Periphery Blood

Periphery blood mononuclear cells (PBMCs) were isolated using a lyse-then-wash step as described previously [25]. Basically, a total of 20 ml venous blood was collected directly into a heparinized tube and diluted with sterile pH 7.2 phosphate-buffered saline (PBS) at room temperature. The PBMCs were then isolated by density gradient centrifugation using Ficoll-Paque Plus (MP Biomedicals, Santa Ana, CA, USA). The acquired PBMCs were washed twice with PBS and resuspended at 1 × 10^7^ cells/ml for culture.

### 2.3. Flow Cytometry Assay

The flow cytometry assays were performed as described previously [39]. All the fluorescence-conjugated antibodies were purchased from BD Biosciences (Ashland, OR, USA). Briefly, PBMCs from healthy donors and patients were stained with the antibodies as indicated, followed by flow cytometry analysis with a BD Biosciences Digital LSR II (BD Biosciences, Franklin Lakes, NJ, USA). Data were analyzed using FlowJo software (Tree Star Inc., Ashland, OR, USA).

### 2.4. Enzyme-Linked Immunosorbent Assay (ELISA)

IFN-*γ*, TNF-*α*, IL-2, IL-4, IL-6, IL-10, IL-17A, and TGF-*β* were detected by using human ELISA kits from BD Bioscience (Franklin Lakes, NJ, USA) according to the manufacturer's instructions. The concentrations of serum IL-21 in MG patients and healthy donors were determined by ELISA using the human IL-21 ELISA kit (R&D Systems, Minneapolis, MN, USA) according to the manufacturer's instructions. Briefly, individual serum at 1 : 4 dilutions were subjected to ELISA analysis, and the concentrations of serum cytokines in individual samples were quantified by reference to standard curves. Determinations were performed in duplicate and results were expressed as pg/ml.

### 2.5. Purification and Sorting of Human Treg Cells

Human Treg cells and Teff cells were purified from the whole blood of healthy human donors. Firstly, CD4^+^ T cells were enriched using RosetteSep Human CD4^+^ T Cell Enrichment Cocktail (STEMCELL Technologies, Vancouver, Canada). CD4^+^ cells were stained with anti-CD4, anti-CD25, and anti-CD127. Treg cells were gated on the CD4^+^CD25^+^CD127^−^ population, and Teff cells were gated on the CD4^+^ CD25^−^CD127^+^ population. All the fluorescence-conjugated antibodies were purchased from BD Biosciences (Ashland, OR, USA).

### 2.6. Generation of Human DCs

CD14^+^ monocytes were isolated from PBMC by using CD14 magnetic beads (Miltenyi Biotec, Gladbach, Germany) and the purity was >98%. Monocytes were cultured in RPMI-1640 medium containing 10% fetal calf serum for 6 days in the presence of cytokines GM-CSF (1000 IU/10^6^ cells) and IL-4 (500 IU/10^6^ cells) to obtain DCs and were used for subsequent experiments.

### 2.7. Coculture of DCs with CD4^+^ T Cells

PBMC-derived DCs were extensively washed and were cocultured with 1 × 10^5^ CD4^+^ T with a 1 : 10 ratio in 96-well U-bottom plates as reported previously. Cocultures were maintained for 4 days and CTLA-4^+^ Tregs were analyzed by flow cytometry (LSR II; BD Biosciences) by using a combination of CD4, CD25, FOXP3, and CTLA-4 antibodies.

### 2.8. Bisulfite Sequencing

Bisulfite sequencing was performed as described previously [40]. Genomic DNA was prepared using an AllPrep DNA Mini Kit (Qiagen, Hilden, Germany). DNA methylation was detected in T cell subsets at the promoter region of CTLA-4. DNA was bisulfite treated using an EpiTect Plus Bisulfite Conversion Kit (Qiagen, Hilden, Germany) according to the manufacturer's instructions. PCR products were purified and sequenced. DNA methylation analysis was carried out using quantification tool for methylation analysis, and methylation was determined at each CpG dinucleotide [41].

### 2.9. Real-Time PCR

Real-time PCR analysis was performed as described previously [42]. Briefly, total RNA was isolated from cells with an RNeasy Mini Kit (Qiagen, Hilden, Germany) according to the manufacturer's instructions. Total RNA from each sample was reverse transcribed with oligo(dT) 20 using SuperScript III Reverse Transcriptase (Invitrogen, Camarillo, CA, USA) followed by real-time PCR. Primers for *CTLA-4* were described as previously [43]. Real-time PCR was performed with SYBR Green PCR Master Mix reagents using an ABI Prism 7700 Sequence Detection System (Applied Biosystems, Foster City, CA, USA).

### 2.10. Statistical Analysis

Data analysis was performed by using SPSS version 16.0. The data with normal distribution are presented as the mean ± standard deviation (SD). To reduce the error of test results at different time points, the patients and healthy donors at the same time point were compared using paired samples *T* test. For the comparison between different treatment groups, the data were analyzed by one-way ANOVA and comparison between the two groups was carried out with a Bonferroni/Dunnett multiple comparison test. A *P* value of less than 0.05 was considered statistically significant.

## 3. Results

### 3.1. Profile Comparison of CD4^+^ T Subset Cells in MG Patients and Healthy Donors

**Figure 1:**
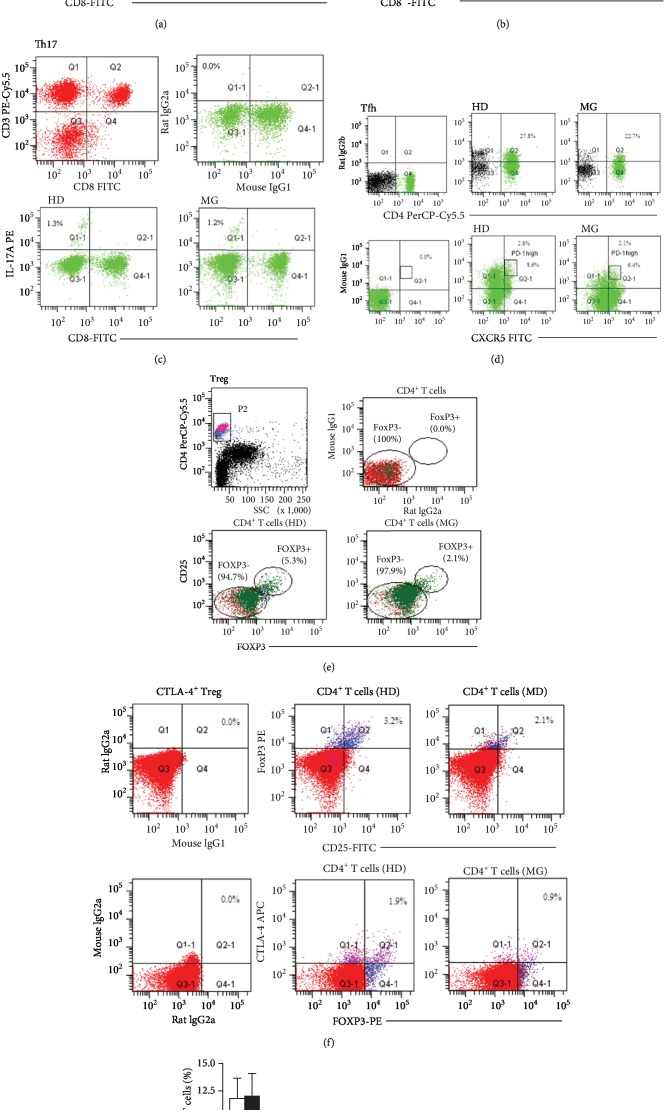
Phenotypic profiles of CD4^+^ T cell subsets reveal underrepresentation of Treg and CTLA-4^+^ Treg in MG patients. Representative FACS analysis of PBMCs from healthy donors and untreated MG patients was shown based on cell surface markers and/or intracellular cytokines for CD4^+^IFN-*γ*^+^ Th1 cells (a), CD4^+^IL-4^+^ Th2 cells (b), CD4^+^IL-17A^+^ Th17 cells (c), CD4^+^CXCR5^+^PD-1^high^ Tfh cells (d), CD4^+^CD25^+^FOXP3^+^ Treg cells (e) and CD4^+^CD25^+^FOXP3^+^CTLA-4^+^ Treg cells (f). G. Comparison of the mean frequency of CD4^+^ T cell subsets based on FACS analysis using the aforementioned markers in healthy donors (*n* = 59) and untreated MG patients (*n* = 39). Data are expressed as mean ± SD. ^∗^*P* < 0.05.

We conducted a comprehensive flow cytometry study to determine the frequency of CD4^+^ T cell subsets including Th1, Th2, Th17, Tfh, Treg, and CTLA-4^+^ Treg in the PBMCs of 59 healthy donors and 40 untreated MG patients. We used the following markers to identify each CD4^+^ T cell subset: Th1 (CD4^+^IFN-*γ*^+^) (Figure 1(a)), Th2 (CD4^+^IL-4^+^) (Figure 1(b)), Th17 (CD4^+^IL-17A^+^) (Figure 1(c)), Tfh (CD4^+^CXCR5^+^PD-1^high^) (Figure 1(d)), Treg(CD4^+^CD25^+^FOXP3^+^) (Figure 1(e)), and CTLA-4^+^ Treg (CD4^+^CD25^+^ FOXP3^+^CTLA-4^+^) (Figure 1(f)). The results are summarized in Figure 1(g) and indicated that the mean frequency of Treg cells and CTLA-4^+^ Treg in MG patients (2.35 ± 0.42% and 0.91 ± 0.31%, respectively) was significantly lower than that in healthy donors (4.70 ± 0.76% and 1.90 ± 0.63%, respectively). In contrast, no significant difference was observed in the frequency of other T cell subsets including Th1 (9.7 ± 2.1% in MG vs. 9.1 ± 1.8% in HD), Th2 (0.62 ± 0.28% in MG vs. 0.45 ± 0.17% in HD), Th17 (1.23 ± 0.31% in MG vs. 1.30 ± 0.44% in HD), and Tfh (2.14 ± 0.47% in MG vs. 2.80 ± 0.64%).

### 3.2. The Profile of Secreted Cytokines in MG Patients

**Figure 2:**
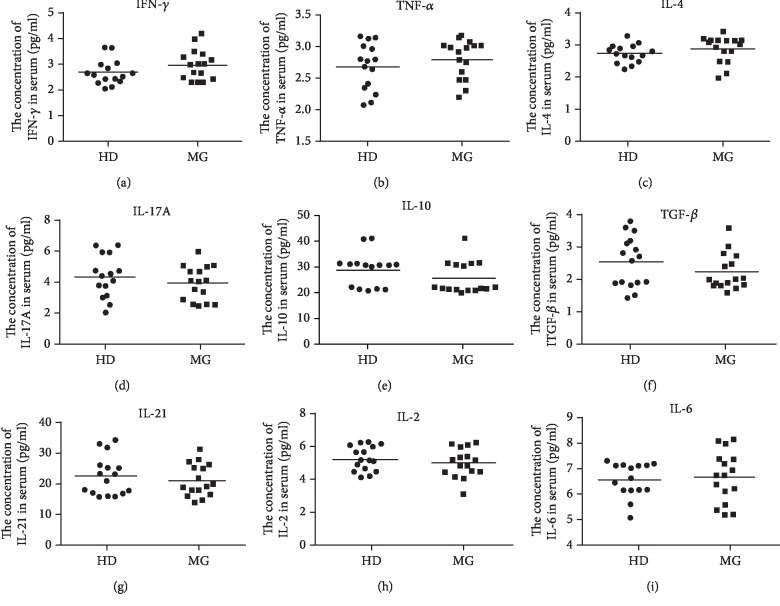
Cytokine profiles of CD4^+^ T cell subset are unchanged in MG patients. The level of cytokines secreted by PBMC from MG patients (*n* = 16) and healthy donors (*n* = 16) was measured by ELISA including Th1-associated IFN-*γ* (a) and TNF-*α* (b), Th2-secreted IL-4 (c), Th17-specific IL-17A (d), Treg-associated IL-10 (e) and TGF-*β* (f), Tfh-secreted IL-21 (g), and another two T cell-secreted cytokines IL-2 (h) and IL-6(i). Each data point represents an individual subject. The horizontal lines represent the average level.

We applied ELISA assay to measure the serum concentrations of a range of cytokines in the MG patients without therapy and healthy donors: IFN-*γ*, TNF-*α*, IL-4, IL-17A, IL-10, TGF-*β*, IL-21, IL-2, and IL-6. The results in Figure 2 indicated the level of IFN-*γ*, TNF-*α*, and IL-4 was higher in MG patient, but statistics revealed no significant difference from healthy donors. The level of IL-17A, IL-21, TGF-*β*, and IL-10 in MG patients was insignificantly lower than healthy donors, respectively. The level of IL-2 and IL-6 remained relatively unchanged in MG patients. We also quantified the mRNA level of each cytokine by qRT-PCR and the results were compatible with the ELISA assay (data not shown). The majority of cytokine levels measured in our study are consistent with previously published reports that showed no significant change of cytokines secreted by PBMC were detected between MG patients and healthy donors [44].

### 3.3. Dynamic Profiling of Treg Cells in MG Patients in Response to IVIg Therapy

**Figure 3:**
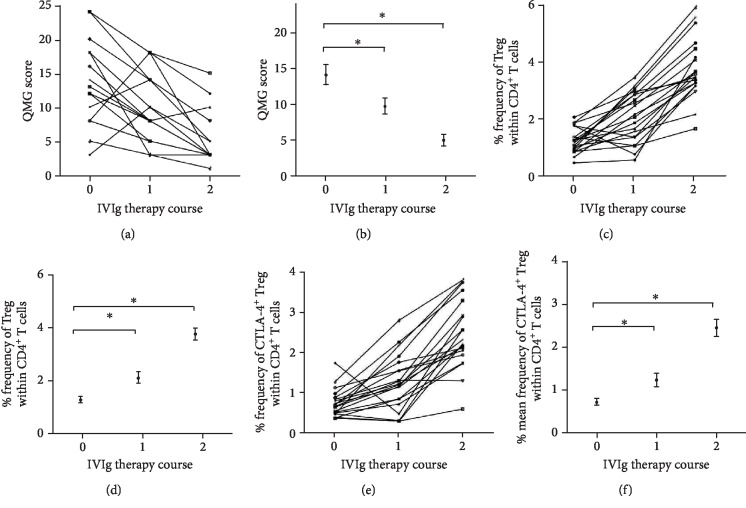
Dynamic changes of regulatory CD4^+^ T cells in MG patients and their QMG scores during IVIg therapy demonstrated a positive correlation between IVIg therapeutic effect and frequency of Tregs and CTLA-4^+^ Tregs. The individual (a) and mean (b) QMG scores were determined for MG patients (*n* = 20) during the two courses of IVIg therapy. The individual and mean frequency of CD4^+^CD25^+^FOXP3^+^ Treg cells (c, d) and CD4^+^CD25^+^FOXP3^+^CTLA-4^+^ Treg cells (e, f) were measured by FACS analysis of PBMCs from IVIg-treated MG patients (*n* = 20).

Intravenous immunoglobulin (IVIg) is widely used in the treatment of MG patients. In this study, 20 out of 39 MG patients received two courses of intravenous immunoglobulin (IVIg) therapy with each course consisting of a dose of 0.4 mg/kg/day for five consecutive days. The therapeutic effects of IVIg, evaluated by dynamic profiling of individual and mean QMG score during IVIg therapy (Figures 3(a) and 3(b)), indicated 16 out of 20 (80%) were relieved after one course of IVIg treatment and 19 out of 20 were significantly improved after two courses of IVIg treatment. The frequency of circulating Treg (CD4^+^ CD25^+^ FOXP3^+^) (Figures 3(c) and 3(d)) and CTLA-4^+^ Treg (CD4^+^CD25^+^FOXP3^+^CTLA-4^+^) (Figures 3(e) and 3(f)) was significantly increased by IVIg. The dynamic change of the frequency of Treg/CTLA-4^+^ Treg cells and clinical symptom by IVIg therapy, and the inverse relationship between them, suggested a correlation between immunologic disorder in MG patients and peripheral Tregs and CTLA-4^+^ Treg population.

### 3.4. IVIg Therapy Induces Treg and CTLA-4^+^ Treg Cells through DCs in MG

**Figure 4:**
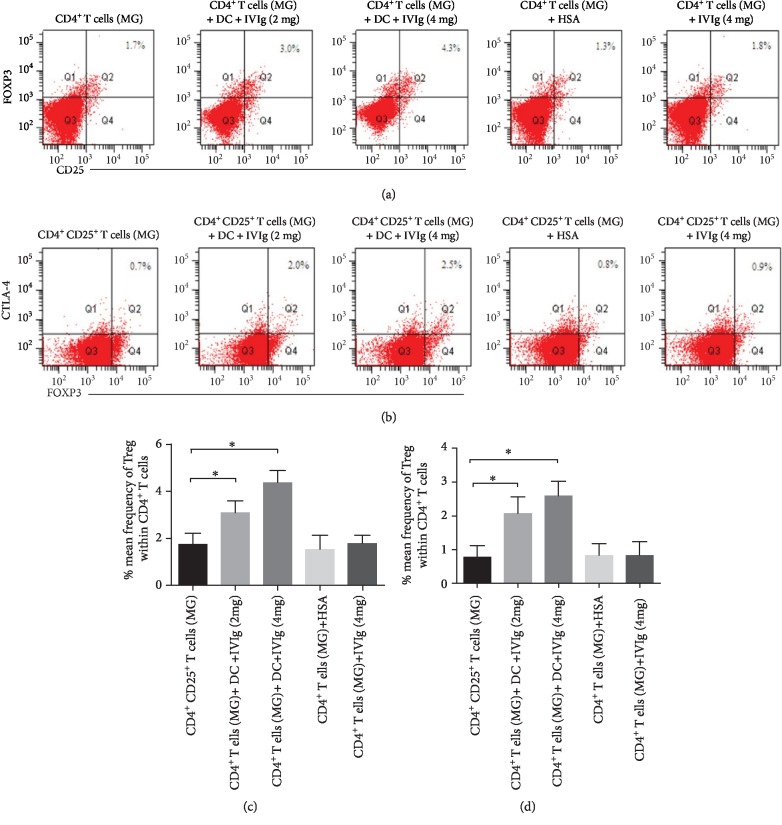
IVIg therapy induces Treg and CTLA-4^+^ Treg cells through DCs in MG. Monocyte-derived DCs from MG patients were treated with vehicle, 2 mg/ml or 4 mg/ml IVIg and then cocultured with CD^4+^ T cells from PBMC of MG patients for 4 days. Treg cells (CD4^+^CD25^+^FOXP3^+^) and CTLA-4^+^ Treg cells (CD4^+^CD25^+^FOXP3^+^CTLA-4^+^) were then detected by flow cytometry (a, b). At least three independent experiments were performed and statistical significance was determined by *T* test (c, d). Data are expressed as mean ± SD. ^∗^*P* < 0.05.

The beneficiary effects of IVIg on MG patients and the inverse relationship between circulating Tregs/CTLA-4^+^ Tregs and disease severity of MG patients prompted us to investigate the mechanisms of expansion of Tregs/CTLA-4^+^ Tregs by IVIg. Because induction of Tregs (CD4^+^CD25^+^FOXP3^+^) in the periphery of healthy donors has been shown to involve the presence of antigen-presenting cells such as dendritic cells (DCs) [45], we sought to determine whether IVIg-induced expansion of circulating Tregs in MG patients through DCs as well. We treated six-day-old monocyte-derived DCs from MG patients with IVIg at a dose that is compatible with that used in the IVIg therapy (2 mg/ml or 4 mg/ml). The DCs were then extensively washed and cocultured with CD4^+^ T cells from PBMC of MG patients for 4 days. We assessed the expression of Tregs (CD4^+^CD25^+^FOXP3^+^) and CTLA-4^+^ Tregs (CD4^+^CD25^+^FOXP3^+^CTLA-4^+^) before or after IVIg treatment by flow cytometry. Compared with untreated DCs or HSA-treated DCs, IVIg-treated DCs significantly increased the expression of Tregs and CTLA-4^+^ Tregs and in a dose-dependent manner (Figures 4(a)–4(d)). In contrast, incubation of CD4^+^ T cells with IVIg prior to coculture with DCs did not increase the expression of Tregs or CTLA-4^+^ Tregs (Figures 4(c) and 4(d)), suggesting that IVIg-induced expansion of Tregs and CTLA-4^+^ Tregs in MG patients through modulation on DCs and the ability of DCs to induce Tregs and CTLA-4^+^ Tregs is not altered in MG patients.

### 3.5. Defective CTLA-4 in MG Treg Is Associated with Diminished Transcription Activity of CTLA-4 Gene Promoter following Methylation

**Figure 5:**
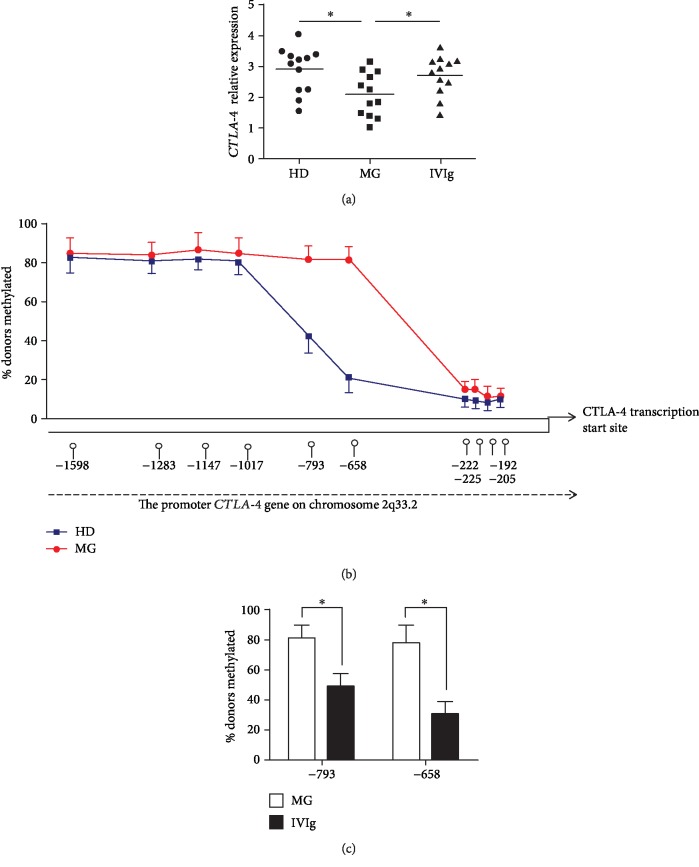
Quantitative analysis of *CTLA-4* mRNA in Treg cells from myasthenia gravis patients (MG) and healthy donors (HD). (a) Total RNA was extracted from sorted Treg cells of MG and HD and then real-time PCR was performed to measure *CTLA-4* mRNA level. (b) Genomic DNA was prepared from sorted Treg cells and methylation status within *CTLA-4* promoter was detected. The methylation percentage within *CTLA-4* promoter in Treg cells from HD and MG (*n* = 10) was shown. (c) The methylation percentage at -658 and -793 CpGs in Treg cells from MG and MG with IVIg therapy (*n* = 12) is shown. Data are expressed as mean ± SD. ^∗^*P* < 0.05.

Having established the role of CTLA-4 in MG pathology and therapy, we sought to investigate the mechanism underlying the defects in CTLA-4 in MG patients. We first determined whether the reduction in CTLA-4 expression occurs at a transcriptional level by comparing the mRNA level of CTLA-4 in the Tregs in MG patients and healthy donors. The results indicated that the expression level of transcripts encoding the total *CTLA-4* was significantly reduced in MG patients and IVIg therapy restored the expression of *CTLA-4* (Figure 5(a)).

Because DNA methylation followed by transcriptional silencing has been recognized as an epigenetic mechanism in maintaining T cell function and altered DNA methylation patterns have been implicated in autoimmunity [40, 46], we hypothesized that altered methylation within CTLA-4 gene promoter can attenuate CTLA-4 gene expression in MG Tregs. We compared the methylation state of CpGs within CTLA-4 gene promoter region in Tregs of MG patients and healthy donors and observed increased methylation at -658 and -793 CpGs (position relative to the ATG start codon) in MG Tregs (Figure 5(b)). Interestingly, IVIg therapy significantly reduced the methylation level at these two sites in MG patients (Figure 5(c)). Taken together, these results indicated that *CTLA-4* exhibited lower expression level due to promoter methylations in MG patients; however, IVIg therapy restored *CTLA-4* expression through reducing methylation level.

## 4. Discussion

Myasthenia gravis (MG) is a CD4^+^ T cell-dependent autoimmune disease and intravenous immunoglobulin (IVIg) has been the mainstay of immunotherapeutic therapy. The subsets of CD4^+^ T cells involved in this disease and IVIg therapy, however, remain to be investigated [12, 36]. Through this study, we have established the roles of CTLA-4^+^ Treg cells in MG and their dynamic profiling in MG patients with IVIg therapy. Initially, we demonstrated that CTLA-4^+^ Treg cells exhibited a lower level than those of healthy donors and IVIg therapy expands CTLA-4^+^ Treg cells in MG patients. Mechanistically, we found that IVIg-induced CTLA-4^+^ Treg cell expansion depends on the modulation of dendritic cells. Finally, hypermethylation of the CTLA-4 promoter was observed in MG patients and IVIg reversed this effect. Taken together, these findings suggest that the key role of CTLA-4 in functionally defected Treg cells and may provide a potential approach for the therapy of this disease.

Cytotoxic T lymphocyte antigen- (CTLA-) 4 is an inhibitory relative of the T cell costimulatory molecule CD28. While CD28 signaling promotes T cell activation, CTLA-4 serves an immune regulatory function, suppressing the T cell response. Widespread recognition of the importance of the CTLA-4 pathway came about when mice deficient of the CTLA-4 gene were found to exhibit dysregulated T cell immunity resulting in tissue infiltration and death around 3 wk of age [21, 32, 47]. Increasing reports of allelic association between specific polymorphisms of CTLA-4 gene with various autoimmune diseases support the idea that the CTLA-4 region is an important locus for autoimmune disease in general. Several studies have suggested the association between mRNA level and specific polymorphisms of CTLA-4 with MG. The role of CTLA-4 and its related T cells in MG, however, remain to be investigated. [35, 48–50]. Here, we measured the frequencies of CD4^+^ T cell subsets in MG patients and found that the mean frequency of Treg cells and CTLA-4^+^ Treg in MG patients was significantly lower than that in healthy donors. As for other CD4^+^ T cell subsets, there is no significant difference between in healthy donors and MG patients. This result indicates that CTLA-4 is involved in the pathogenesis of MG.

Intravenous immunoglobulin (IVIg) is extensively used in the treatment of autoimmune and inflammatory diseases. Although IVIg therapy has been used for close to 3 decades, the mechanism of action is incompletely understood [36, 37]. Treg cells play a critical role in the maintenance of immune tolerance and prevention of autoimmunity. Deficiency of Tregs or their defective functions lead to autoimmune diseases, whereas Treg expansion and function regains are associated with recovery from autoimmune diseases. IVIg expands FOXP3^+^ Treg cells mediated by dendritic cells (DCs) in autoimmune diseases [51, 52]. CTLA-4 plays a critical role in the function of Tregs and it incorporates with FOXP3 to represent the complete suppression function of Tregs. As for MG, a recent study suggests that immunosuppressive treatment leads to Treg subpopulation change in MG patients. This study has well established the association of immunosuppressive treatments with Treg subpopulation recovery [26]. As we know, there is no data suggesting the association between CTLA-4 and IVIg therapy in autoimmune diseases [53, 54]. Here, we found that IVIg therapy expanded CTLA-4^+^ Tregs in MG patients. We firstly established the possible association between CTLA-4^+^ Treg expansion and IVIG therapy. Since CTLA-4 plays a critical role in MG recovery, it would be a very important strategy to increase CTLA-4 of Treg in the therapy of MG and other autoimmune diseases. Considering the limitation of patient sample size in this study, we will perform our further study using more MG patient samples to confirm the preliminary data.

DNA methylation plays a critical role in gene expression regulation through establishing and maintaining the DNA methylation status in gene promoters. DNA methylation mainly occurs in the CpG islands of gene promoters and it decreases gene expression by inhibiting the recruitments of transcriptional factors. It has been shown that CTLA-4 promoter methylation involved the pathology of rheumatoid arthritis [40, 46]. This led us to postulate that DNA methylation of *CTLA-4* promoter also occurs in MG patients. As a result, hypermethylation at -658 and -793 CpGs of *CTLA-4* promoter was observed in MG patients. Importantly, IVIg therapy reversed the hypermethylation of *CTLA-4* promoter. A recent study suggests that the hypermethylation of *CTLA-4* promoter is associated with the pathogenesis of MG [55]. This and our studies indicated that it may be an alternative therapy strategy to inhibit hypermethylation of *CTLA-4* promoter in MG patients. Another study also reported that IVIg treatment leads to methylation alterations of inflammatory immune-associated genes in Kawasaki disease. An interesting but challenging question raised by these results is what causes the methylation change to occur and how IVIg therapy reverses this effect in the *CTLA-4* gene promoter. We attempted to address this mechanistically through RNA-seq in MG patients but we failed to identify a potential factor. We will try other molecular tools as well as performing RNA-seq using more samples.

## 5. Conclusions

In summary, our preliminary data suggest that CTLA-4 may play a role in the disease progress and recovery of MG.

## Figures and Tables

**Table 1:** The clinical characteristics of MG patients.

MGFA class	Number	Age (mean ± SD)	Male/female	IVIg therapy
I	9	39.33 ± 11.24	4/5	0
II	16	37.47 ± 15.21	3/13	6
III	9	43.33 ± 15.46	5/4	9
IV	5	45.60 ± 11.06	1/4	5
Total	39	39.34 ± 14.37	13/26	20

## Data Availability

The data used to support the findings of this study are available from the corresponding authors upon request.
